# Projected Affinity Values for Nyström Spectral Clustering

**DOI:** 10.3390/e20070519

**Published:** 2018-07-10

**Authors:** Li He, Haifei Zhu, Tao Zhang, Honghong Yang, Yisheng Guan

**Affiliations:** 1 Department of Electromechanical Engineering, Guangdong University of Technology, Guangzhou 510006, China; 2 Department of Computing Science, University of Alberta, Edmonton, AB T6G 2R3, Canada; 3 School of Automation, Northwestern Polytechnical University, Xi’an 710072, China

**Keywords:** Nyström approximation, out-of-sample, empirical affinity, machine learning

## Abstract

In kernel methods, Nyström approximation is a popular way of calculating out-of-sample extensions and can be further applied to large-scale data clustering and classification tasks. Given a new data point, Nyström employs its empirical affinity vector, *k*, for calculation. This vector is assumed to be a proper measurement of the similarity between the new point and the training set. In this paper, we suggest replacing the affinity vector by its projections on the leading eigenvectors learned from the training set, i.e., using $k^{*}=\sum_{i=1}^{c}k^{T}u_{i}u_{i}$ instead, where $u_{i}$ is the *i*-th eigenvector of the training set and *c* is the number of eigenvectors used, which is typically equal to the number of classes designed by users. Our work is motivated by the constraints that in kernel space, the kernel-mapped new point should (a) also lie on the unit sphere defined by the Gaussian kernel and (b) generate training set affinity values close to *k*. These two constraints define a Quadratic Optimization Over a Sphere (QOOS) problem. In this paper, we prove that the projection on the leading eigenvectors, rather than the original affinity vector, is the solution to the QOOS problem. The experimental results show that the proposed replacement of *k* by $k^{*}$ slightly improves the performance of the Nyström approximation. Compared with other affinity matrix modification methods, our $k^{*}$ obtains comparable or higher clustering performance in terms of accuracy and Normalized Mutual Information (NMI).

## 1. Introduction

Over the last decade, clustering algorithms have played an important role in signal processing and data mining in applications such as community detection [1,2], image segmentation [3,4,5], remote image processing [6], big data network clustering [7] and subspace analysis [8]. Cluster analysis attempts to partition data points into disjoint groups such that data points that correspond to the same label are similar and the cross-group similarity is small.

In large-scale clustering, Nyström approximation is a common choice for approximating the eigensystem of a large matrix. Given a training set and its eigensystem, to perform Nyström approximation, one calculates the similarity values between the training set and the remaining data and uses the resulting similarity matrix to approximate the eigenvectors of the remaining data points.

A fundamental problem in Nyström approximation is the measurement of the similarity between a training set and its remaining data points. Given a new data point and a Gaussian kernel parameter σ, most methods employ the empirical affinity vector, *k*, to evaluate the links between this new point and the training set:

(1) $$k_{i}=\exp\left(-∥x_{new}-x_{i}{∥}^{2}/2\sigma^{2}\right)$$

where $k_{i}$ is the *i*-th element in *k*, $x_{new}$ denotes the new point and $x_{i}$ is the *i*-th point in the training set.

Despite its broad applications in kernel approximation, in this paper, we still focus our attention on the construction of *k*. We begin our argument with the following question: Given the eigensystem of a training set, should we evaluate the similarity by the Euclidean distance, which is isometric? With the training set eigensystem in hand, we determine the underlying structure of the data or, more specifically, the manifold on which the data points lie. Thus, we prefer to evaluate similarities on the manifold, instead of using an isometric metric, such as the Euclidean distance. In this paper, we analyse the construction of *k*, which comports with the learned manifold. Our goal in this paper is to construct a new *k* for Nyström approximation and, consequently and ultimately, improve the clustering performance.

Many works on the modification of *k* have been proposed in recent years. Sparse representation is one of the most promising techniques in the literature. Sparse representation describes data in a low-dimensional subspace in which data are self-expressive. Data in such a subspace can be efficiently encoded as a linear or affine combination of other points. Sparse spectral clustering and its variants employ various cost functions that differ in their sparsity measurement and constraints.

**Figure 1:**
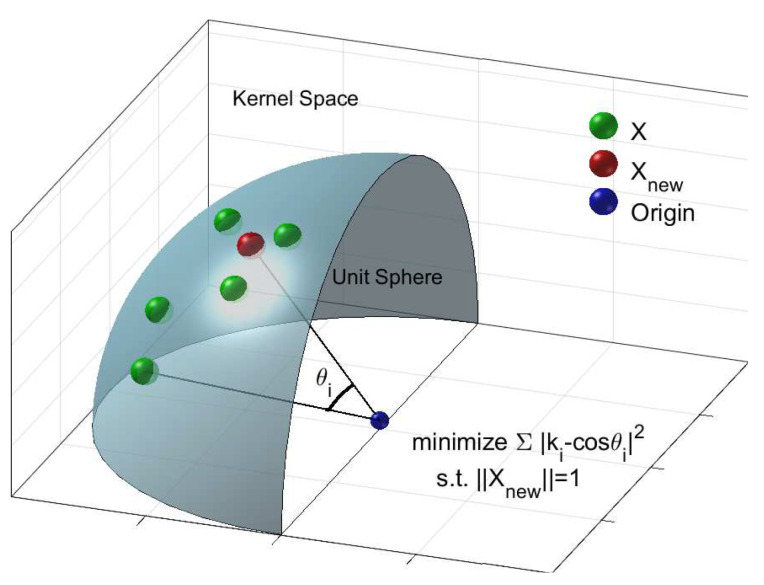
Given the “empirical” affinity vector *k*, we consider the construction of the optimal $k^{*}$ to be the solution that satisfies the following: (a) its corresponding kernel space data point $X_{new}$ minimizes $∥k-X_{new}^{T}{X∥}_{F}$, and (b) $X_{new}$ is on the unit sphere that is defined by the Gaussian kernel. Notice that since *X* is on the unit sphere in kernel space, $X_{i}^{T}X_{j}=\cos\left(\theta_{ij}\right)$ holds for any pair $X_{i}$ and $X_{j}$ with corresponding angle $\theta_{ij}$. Best viewed in colour.

In this paper, we propose replacing *k* with its projections on the leading eigenvectors: $k^{*}=\sum_{i=1}^{c}k^{T}u_{i}u_{i}$. As our motivation, we consider the *k*-construction as the optimal solution that satisfies the following: (a) its corresponding kernel space data point $X_{new}$ minimizes $∥k-X_{new}^{T}{X∥}_{F}$, and (b) $X_{new}$ is on the unit sphere defined by the Gaussian kernel, where *X* stands for the training points in the kernel space. The cost function guarantees that the optimal $k^{*}$ is close to *k* and the constraint ensures that $X_{new}$ is on the unit sphere defined by the Gaussian kernel. We illustrate our motivation in Figure 1.

Our contributions include the following:

- We show that in Nyström approximation, the projections of *k* on the leading training set eigenvectors are more suitable for clustering.
- The projections, entitled $k^{*}$ in this paper, are the solution to the Quadratic Optimization Over a Sphere (QOOS) problem defined by Nyström approximation.
- Experimental results show that replacing *k* with $k^{*}$ generates slight improvements on clustering.

The rest of this paper is organized as follows: Section 2 reviews related works in large-scale data spectral clustering. Our algorithm is described in Section 3. Section 4 shows the experimental results on several real-world datasets. We present our conclusions in Section 5.

## 2. Related Works

The spectral clustering problem is identical to solving for the eigenvectors of a kernel matrix $K\in R^{n\times n}$, which is generated to reveal similarities among data. To speed up the eigenvector solution on *K*, Nyström-based methods [9] are frequently used. The basic idea of Nyström is to sample various data points and construct the low-rank approximation to *K* from the sampled data points. In addition to Nyström, in the subspace clustering community, building the affinity matrix *K* correctly is a fundamental problem. The intrinsic characteristics of the dataset, such as sparsity or nearest neighbours, are employed to modify the affinity matrix *K*. In this section, we categorize the related works into two classes: (a) Nyström methods and (b) subspace clustering. Nyström is the most popular method in approximating eigenvectors in spectral clustering. Subspace clustering, on the other hand, is a general topic that covers spectral clustering and applies Nyström for acceleration.

### 2.1. Nyström

Nyström is an efficient way to generate a low-rank representation of the desired kernel matrix of a large dataset. Without loss of generality, the columns and rows of *K* can be arranged according to the selected columns and:

(2) $$K=\left[\begin{array}{cc} K_{S} & K_{21}^{T}\\ K_{21} & K_{22} \end{array}\right],E=\left[\begin{array}{c} K_{S}\\ K_{21} \end{array}\right]$$

where $K_{S}$ is the similarity matrix among the sampled data, $K_{21}$ is the similarity matrix of the remaining data to the sample set and $K_{22}$ the matrix of similarities among the remaining data.

Nyström uses an m×m matrix $K_{S}$ and an n×m matrix *E* to approximate *K* by:

(3) $$K\approx \tilde{K}=EK_{S}^{+}E^{T}$$

and approximates the leading *l* eigenvalues $\Lambda_{l}$ and eigenvectors $V_{l}$ by:

(4) $${\tilde{\Lambda }}_{l}=\frac{n}{m}\Lambda_{S,l}$$

(5) $${\tilde{V}}_{l}=\sqrt{\frac{m}{n}}EV_{S,l}{\left(\Lambda_{S,l}\right)}^{-1}$$

where *m* is the size of the sample set, $K_{S}^{+}$ represents the Moore–Penrose pseudo-inverse of $K_{S}$ and $\Lambda_{S,l}$ are the leading *l* eigenvalues of $K_{S}$ with the corresponding eigenvectors $V_{S,l}$.

Given the desired number of classes *l*, the corresponding Laplacian matrix should be of rank *l*. Nie et al. [10] accounted for the rank-*l* constraint by minimizing a cost function, which is composed of two parts: the first part ensures that the new affinity matrix is close to the original one, and the second part enforces the rank-*l* constraint. A similar approach is proposed in [11], where adaptive neighbours, instead of the nearest neighbours, were linked in *S*. Recently, Langone and Suykens [12] represented spectral clustering in a weighted kernel PCA formulation, which is well defined in [13]. To reduce the time cost, Zhu et al. [14] proposed Balanced *k*-means based Hierarchical *k*-means (BKHK), which adopts a balanced binary tree, thereby reducing the training computational complexity to O(nlog(m)di), where *d* is the dimension of the input data and *i* here is the iteration BKHK stopped. A dense similarity matrix always requires $O(n^{2})$ in pairwise similarity calculations. Luo et al. [15] estimated the sparse similarity matrix using *k*-means accumulative consensus, which is locality-preserving and of low complexity. Langone et al. [16] proposed an entropy-based Incomplete Cholesky Decomposition (ICD) method to handle the large-scale data clustering. The main idea in [16] is to approximate the Laplacian matrix by ICD and efficiently solve the eigensystem of the approximated Laplacian matrix iteratively.

Most existing Nyström methods focus on a new structure in approximating eigenvectors of *K*, e.g., [12] with kernel PCA and [14] of BKHK in sampling. Our proposed method, however, aims to modify *K*, which is the input to Nyström approximation. Thus, we consider our method as a pre-processing benefiting the follow-ups, such as sampling [14,17] or approximating [16].

### 2.2. Subspace Clustering

Another popular way to modify *k* is subspace clustering, in which different approaches are proposed for evaluating similarity values among data points. The affinity matrix is constructed by using the typical Gaussian kernel or another local-information-based method, which may not be a good choice for subspace clustering. Therefore, several affinity matrix construction methods have been proposed in recent years.

Sparse Subspace Clustering (SSC) [18] searches for a sparse representation matrix *C* as:

(6) $${\min∥C∥}_{1}\;\;\;\;s.t.\;\;\;\;Y=YC,\;\;diag\left(C\right)=0$$

where *Y* is the data matrix. It is proven that the optimal solution *C* by SSC satisfies the block diagonal property when the subspaces are independent. Despite its advantages in clustering a dataset with a highly complex structure, SSC may suffer from the so-called “graph connectivity” issue [19]. The $l_{1}$-norm in SSC ensures not only the sparsity in inter-class affinities, but also the inner-class similarities; in the latter case, we typically prefer a dense and well-linked similarity matrix for clustering. Similar to SSC, different cost functions are introduced for *C*. Other than the sparsity cost, i.e., the $L_{1}$-norm in SSC, Least Squares Regression (LSR) [20] minimizes the Frobenius norm of the representation matrix *C*:

(7) $${\min∥C∥}_{F}\;\;\;\;s.t.\;\;\;\;Y=YC,\;\;diag\left(C\right)=0$$

LSR tends to group highly correlated data together.

In addition to SSC and LSR, similarity by neighbourhood evaluation is a popular *K*-construction approach. Locally Linear Representation (LLR) [21] seeks to solve the following optimization problem for each data point $x_{i}$:

(8) $$\min∥x_{i}-D_{i}c_{i}{∥}_{2}^{2}\;\;\;\;s.t.\;\;\;\;1^{T}c_{i}=1$$

where $c_{i}$ is the coefficient of $x_{i}$ relative to its neighbours and $D_{i}$ consists of the Euclidean distances $x_{i}$ to its *k* nearest neighbours.

## 3. Projections on Leading Eigenvectors as the Solution to the QOOS Problem

There is a critical issue that remains largely unresolved in the literature: given an affinity vector *k* from a Gaussian-kernel-mapped new point, is there a corresponding point $X_{new}$ in the kernel space such that $X^{T}X_{new}=k$ and $∥X_{new}∥=1$? The first equality ensures that $X_{new}$ is the corresponding point to *k* and the second one ensures that $X_{new}$ is on the unit sphere specified by the Gaussian kernel. In this section, we present our main result: the projection of *k* on the leading eigenvectors *u*, which is calculated as $k^{*}=\sum_{i=1}^{c}k^{T}u_{i}u_{i}$, is a solution to the two equalities, where *c* is the user-assigned number of classes in clustering.

### 3.1. Quadratic Optimization Over a Sphere Problem

Instead of focusing on the two equalities directly, we focus on the following substitute question: given *k* and *X*, what is the optimal point $X_{new}^{*}$ that satisfies:

(9) $$\begin{array}{cc} \arg & \min_{X_{new}^{*}}{∥X^{T}X_{new}^{*}-k∥}^{2}\\ & s.t.\;\;∥X_{new}^{*}∥=1 \end{array}$$

Given the fixed value of *k*, it is natural to take this $X_{new}^{*}$ to represent the new data point in the kernel space. Furthermore, if the cost function in Equation (9) reaches zero, then we prove the existence of the kernel-mapped point $X_{new}^{*}$ with respect to *k*.

The study of the optimality of $X_{new}$ is the foundation for many applications. For example, in recent research on explicit feature mapping, Nyström extensions were applied to obtain points in the kernel space explicitly [12]. Then, the EFM of points can be used for further clustering or classification, with the benefit of extremely low computational costs. A detailed analysis on $X_{new}$ provides theoretical guarantees for those methods that employ $X_{new}$. In addition, as discussed in the next section, the projections of *k* on the leading eigenvectors, namely $k^{*}=\sum_{i=1}^{c}k^{T}u_{i}u_{i}$, are good substitutes to *k* in the QOOS problem.

By defining $A=XX^{T}$ and $b=Xk_{new}$, Equation (9) can be represented as:

(10) $$\begin{array}{cc} \arg & \min_{X_{new}^{*}}{\left(X_{new}^{*}\right)}^{T}A\left(X_{new}^{*}\right)-2b^{T}\left(X_{new}^{*}\right)\\ & \;\;\;\;\;\;\;\;s.t.\;\;∥X_{new}^{*}∥=1 \end{array}$$

Equation (10) is a QOOS problem, which was well studied by Hager in [22]. In the coming section, we will see that in Nyström approximation, Equation (10) follows the degenerate case of Lemma 2.2 in [22]. Then we present the closed form of $X_{new}^{*}$.

### 3.2. Degenerate Case of the Gaussian Kernel

As our theoretical foundation, we review the main results in [22] in the following lemma.

**Lemma** **1.** *(Lemmas 2.1, 2.2 in [22], Lemmas 2.4 and 2.8 in [23])*

*Let $A=\Phi \Lambda \Phi^{T}$ be the eigendecomposition of A, where *Λ* is a diagonal matrix with diagonal elements $\lambda_{1}\geq \lambda_{2}\geq ,\ldots ,\geq \lambda_{n}$ and *Φ* consists of corresponding eigenvectors $\phi_{1},\phi_{2},\ldots ,\phi_{n}$. Define $\beta_{i}=b^{T}\phi_{i}$, $\epsilon_{1}=\left\{i:\lambda_{i}=\lambda_{n}\right\}$ and $\epsilon_{+}=\left\{i:\lambda_{i}>\lambda_{n}\right\}$. Then, the vector $\phi =\sum_{i=1}^{n}a_{i}\phi_{i}$ is a solution to Equation (10) if and only if c is chosen in the following way:*

*(a) Degenerate case: If $\beta_{i}=0$ for all $i\in \epsilon_{1}$ and:*

(11) $$\sum_{i\in \epsilon_{+}}\frac{\beta_{i}^{2}}{{\left(\lambda_{i}-\lambda_{n}\right)}^{2}}\leq 1$$

*then $a_{i}=\beta_{i}/\left(\lambda_{i}-\lambda_{n}\right)$ for all $i\in \epsilon_{+}$; $a_{i}$ for $i\in \epsilon_{1}$ are arbitrary scalars that satisfy the condition:*

(12) $$\sum_{i\in \epsilon_{1}}a_{i}^{2}=1-\sum_{i\in \epsilon_{+}}\frac{\beta_{i}^{2}}{{\left(\lambda_{i}-\lambda_{n}\right)}^{2}}$$

*(b) Nondegenerate case: If (a) does not hold, then $a_{i}=\beta_{i}/\left(\lambda_{i}+\mu \right)$ where $\mu >-\lambda_{n}$ is chosen so that:*

$$\sum_{i=1}^{n}\frac{\beta_{i}^{2}}{{\left(\lambda_{i}+\mu \right)}^{2}}=1$$

We will see in this section that our problem is the degenerate case in solving Equation (10). Suppose that we are given the n×n affinity matrix *K* and its eigendecomposition $K=X^{T}X=U\Sigma U^{T}$, where Σ is a diagonal matrix with diagonal elements $\sigma_{1}\geq \sigma_{2}\geq \ldots \geq \sigma_{n}$ and column vectors $u_{1},u_{2},\ldots ,u_{n}$ as the corresponding eigenvectors. Similarly, the eigensystem of $XX^{T}$ is $XX^{T}=\Phi \Lambda \Phi^{T}$, where $\lambda_{i}$ and $\phi_{i}$ are the eigenvalues and eigenvectors, respectively. It is easy to verify that the leading *n* eigenvalues of $XX^{T}$ and $X^{T}X$ are identical, namely $\lambda_{i}=\sigma_{i},i=1,2,\ldots ,n$, with the corresponding eigenvectors $\phi_{i}=Xu_{i}/\sqrt{\sigma_{i}},i=1,2,\ldots ,n$. The remaining m−n eigenvalues of $XX^{T}$ are all equal to zero. Thus,

(13) $$\begin{array}{cc} \Lambda & =diag\left(\lambda_{1},\lambda_{i},\ldots ,\lambda_{m}\right)=diag\left(\underset{n}{\underset{︸}{\sigma_{1},\sigma_{2},\ldots ,\sigma_{n}}},\underset{m-n}{\underset{︸}{0,0,\ldots ,0}}\right)\\ \Phi & =\{\phi_{1},\phi_{2},\ldots ,\phi_{m}\}\\ & =\{\underset{n}{\underset{︸}{Xu_{1}/\sqrt{\sigma_{1}},Xu_{2}/\sqrt{\sigma_{2}},\ldots ,Xu_{n}/\sqrt{\sigma_{n}}}},\\ & \;\;\;\;\underset{m-n}{\underset{︸}{\phi_{n+1},\phi_{n+2},\ldots ,\phi_{m}}}\} \end{array}$$

In many applications such as clustering, the similarity matrix *K* is always a rank-deficient matrix, which indicates that the smallest eigenvalue is zero: $\lambda_{n}=0$.

Let $\gamma_{i}=k^{T}X^{T}\phi_{i}$ or the analogue to $\beta_{i}$ in Lemma 1, $\epsilon_{1}=\left\{i:\lambda_{i}=0\right\}$ and $\epsilon_{+}\left\{i:\lambda_{i}>0\right\}$ where $\lambda_{n}=0$. The degeneracy condition in [22] requires that (a) for all $i\in \epsilon_{1}$, $\gamma_{i}=0$ and (b) $\sum_{i\in \epsilon_{+}}\gamma_{i}^{2}/\lambda_{i}^{2}\leq 1$.

We show in the following theorem that if there exists $X_{new}^{*}$ that satisfies both $X^{T}X_{new}^{*}=k$ and $∥X_{new}^{*}∥=1$, then $X_{new}^{*}$ satisfies the degeneracy condition in Lemma 1.

**Theorem** **1.** If there exists an $X_{new}^{*}$ satisfying $X^{T}X_{new}^{*}=k$ and $∥X_{new}^{*}∥=1$, then the QOOS problem of Equation (9) in the Gaussian kernel Nyström approximation satisfies the degeneracy condition in [22].

**Proof.**  For $i\in \epsilon_{1}$,

$$\begin{array}{cc} \gamma_{i} & =k^{T}X^{T}\phi_{i}={\left(X^{T}X_{new}^{*}\right)}^{T}X^{T}\phi_{i}\\ & ={\left(X_{new}^{*}\right)}^{T}XX^{T}\phi_{i}=0 \end{array}$$

The last equality uses that $\phi_{i}$ is an eigenvector of $XX^{T}$ whose corresponding eigenvalue is zero when $i\in \epsilon_{1}$. Thus, (a) is satisfied.

Let $\theta_{i}$ be the angle between $X_{new}^{*}$ and $\phi_{i}$. For $i\in \epsilon_{+}$,

$$\begin{array}{cc} \frac{\gamma_{i}}{\lambda_{i}} & =\frac{k^{T}X^{T}\phi_{i}}{\lambda_{i}}\\ & =\frac{{\left(X_{new}^{*}\right)}^{T}XX^{T}\phi_{i}}{\lambda_{i}}\\ & ={\left(X_{new}^{*}\right)}^{T}\phi_{i}\\ & =∥X_{new}^{*}∥\cdot ∥\phi_{i}∥\cdot \cos\theta_{i}\\ & =\cos\theta_{i} \end{array}$$

where in the last equality, we use the constraint $∥X_{new}^{*}∥=1$. Therefore, $\sum_{i\in \epsilon_{+}}\gamma_{i}^{2}/\lambda_{i}^{2}=\sum_{i\in \epsilon_{+}}{\left(\cos\theta_{i}\right)}^{2}\leq 1$, where equality holds if and only if the eigenvectors in $\epsilon_{+}$ span the complete kernel space, i.e., $i\in \epsilon_{+}=\left\{1,2,\ldots ,m\right\}$. Thus, (b) is satisfied. ☐

### 3.3. Solution to the QOOS Problem

As shown in Lemma 1, the vector $X_{new}^{*}=\sum_{i=1}^{m}a_{i}\phi_{i}$ is a solution to Equation (9) if *a* is chosen in the following way [22]: (a) for all $\lambda_{i}\neq 0$, or $i\in \epsilon_{+}$, $a_{i}=\gamma_{i}/\lambda_{i}$ and (b) $a_{i}$ for $\lambda_{i}=0$ are arbitrary scalars that satisfy the following condition:

(14) $$\sum_{i\in \epsilon_{1}}a_{i}^{2}=1-\sum_{j\in \epsilon_{+}}a_{j}^{2}$$

Suppose there are *p* non-zero eigenvalues when i=1,2,…,n,

$$\Lambda =diag(\underset{p}{\underset{︸}{\sigma_{1},\sigma_{2},\ldots ,\sigma_{p}}},\underset{n-p}{\underset{︸}{0,0,\ldots ,0}},\underset{m-n}{\underset{︸}{0,0,\ldots ,0}})$$

Recall that $\sum_{j\in \epsilon_{+}}a_{j}^{2}=\sum_{j\in \epsilon_{+}}\gamma_{j}^{2}/\lambda_{j}^{2}$. Then, for i={p+1,p+2,…,n}, we set:

(15) $$a_{s}=a_{i}={\left[\frac{1}{n-p}\left(1-\sum_{j\in \epsilon_{+}}\gamma_{j}^{2}/\lambda_{j}^{2}\right)\right]}^{1/2}$$

or, we set all scalars $a_{i}$, i∈{p+1,…,n}, of the n−p zero eigenvalues to be equal. Then, we have:

(16) $$c=\{\underset{p}{\underset{︸}{\gamma_{1}/\lambda_{1},\gamma_{2}/\lambda_{2},\ldots ,\gamma_{p}/\lambda_{p}}},\underset{n-p}{\underset{︸}{a_{s},a_{s},\ldots ,a_{s}}},\underset{m-n}{\underset{︸}{0,0,\ldots ,0}}\}$$

Finally, we obtain the solution to Equation (9) by:

(17) $$\begin{array}{cc} X_{new}^{*} & =\sum_{i=1}^{m}a_{i}\phi_{i}\\ & =\sum_{i=1}^{p}\gamma_{i}\phi_{i}/\lambda_{i}+\sum_{i=p+1}^{n}a_{s}\phi_{i}+\sum_{i=n+1}^{m}0\cdot \phi_{i}\\ & =\sum_{i=1}^{p}\frac{\gamma_{i}}{\lambda_{i}}\phi_{i}+a_{s}\sum_{i=p+1}^{n}\phi_{i} \end{array}$$

### 3.4. Projections of *k* on the Leading Eigenvectors

In this section, we present our main result that $k^{*}=\sum_{i=1}^{c}k^{T}u_{i}u_{i}$ is a proper affinity vector with corresponding $X_{new}^{*}$ that satisfies Equation (9). Given the closed form of $X_{new}^{*}$ from Equation (17), we want to obtain the affinity vector of $X_{new}^{*}$, namely, $X^{T}X_{new}^{*}$, and determine whether $X^{T}X_{new}^{*}=k$.

Notice that:

(18) $$X^{T}\Phi_{i}=X^{T}Xu_{i}/\sqrt{\lambda_{i}}=\lambda_{i}^{1/2}u_{i}$$

and recall the definition of $\gamma_{i}$, namely, $\gamma_{i}=k^{T}X^{T}\Phi_{i}=k^{T}\lambda_{i}^{1/2}u_{i}$, where in the second equality, we use Equation (18). Then, $X^{T}X_{new}^{*}$ follows:

(19) $$\begin{array}{cc} X^{T}X_{new}^{*} & =\sum_{i=1}^{p}\frac{\gamma_{i}}{\lambda_{i}}X^{T}\Phi_{i}+a_{s}\sum_{j=p+1}^{n}X^{T}\Phi_{j}\\ & =\sum_{i=1}^{p}k^{T}\lambda_{i}^{-1/2}u_{i}\cdot \lambda^{1/2}u_{i}+a_{s}\sum_{j=p+1}^{n}\lambda_{j}^{1/2}u_{j}\\ & =\sum_{i=1}^{p}k^{T}u_{i}u_{i} \end{array}$$

where in the last equality, we use that $\lambda_{j}=0$ for j=p+1,…,n.

Equation (19) shows that the projection of *k* on any non-zero eigenvector, namely, $k_{+}^{*}=\sum_{i=1}^{p}k^{T}u_{i}u_{i}$, is a proper affinity vector with corresponding $X_{new}^{*}$ that satisfies both $∥X^{T}X_{new}^{*}-k_{+}^{*}∥=0$ and $∥X_{new}^{*}{∥}^{2}=1$. Thus, we can replace *k* with $k_{+}^{*}$ in the Nyström approximation.

In clustering tasks, we always use the leading eigenvectors to reveal the underlying structure of the data. For an ideal clustering task, i.e., a task in which the affinity value is one of two data points in the same class and zero for cross-class data points, there are exactly *c* non-zero eigenvalues/eigenvectors, where *c* is the number of classes. For a general clustering task in which affinity values are distorted, we assume that the leading *c* eigenvectors span the proper subspace for clustering. Thus, instead of projecting *k* on all *p* non-zero eigenvectors as shown in Equation (19), we use $k^{*}=\sum_{i=1}^{c}k^{T}u_{i}u_{i}$ in our work. We summarize our $k^{*}$-based clustering approach in Algorithm 1.

**Algorithm 1** Projected Affinity Values (PAVs) for Nyström spectral clustering.**INPUT:** Dataset *x*, Gaussian kernel parameter σ, training set size *s*, number of classes *c* **OUTPUT:** Class labels of data.
Randomly select *s* data points from *x* as the training set and build the affinity matrix *W* of the training set.Eigendecomposition of *W*, $W=\sum_{i=1}^{s}\lambda_{i}u_{i}u_{i}^{T}$.Calculate affinity vector *k* for one testing data point.// *Projections*
$k^{*}=\sum_{i=1}^{c}k^{T}u_{i}u_{i}$
Run NCut on *W* and use $k^{*}$ in the Nyström approximation to obtain the embeddings of the testing data point.Run *k*-means clustering on the embeddings of the entire dataset.


According to Algorithm 1, compared with the traditional Nyström method, we only replace the affinity vector *k* with $k^{*}$ and adopt all other processes. Since both *k* and *u* are also used in Nyström, our construction of $k^{*}$ introduces very few additional operations.

As can be seen from Algorithm 1, the time consumption of our method is very close to that of the standard Nyström. In general, Nyström requires $O(nmd+m^{3}+nm^{2})$ in time complexity, where *n* stands for the volume of the dataset and *m* is the size of the training set; *d* here is the dimension of the input data. In the time complexity of Nyström, the first part O(nmd) stands for the Nyström approximation, the second $O(m^{3})$ refers to the eigen-solver of processing with the training set and the last $O(nm^{2})$ comes from the final embeddings of all *n* data points. In our method, we take the eigenvectors $u_{i}$ of *K* as input, and such eigenvectors $u_{i}$ are also required in the standard Nsytröm. That means we require no additional time cost for our input. Given $u_{i}$, we project *k* on $u_{i}$, and such vector projections take very limited operations.

## 4. Experiment

In this section, the proposed affinity projection method is verified on several real-world datasets. All experiments were carried out in MATLAB R2016b. Our platform for running these experiments was a workstation equipped with sixteen 2.10-GHz CPUs and eight 16-GB RAM.

### 4.1. Competing Methods and Evaluation Metrics

Experiments were executed on several benchmark datasets from the UCI machine learning repository [24], MNIST-8M [25] and EMNIST Digits (training set) [26,27]. Details of the employed datasets are shown in Table 1.

To evaluate the performance of our PAV, we compare it with four popular clustering methods on several benchmark datasets. The competing methods are listed as follows:

- Standard Nyström (Nys), in which we use half of the data for training.
- Sparse Spectral Clustering [18] (SSC), in which we adopt the default settings in the original code.
- Local Subspace Analysis [28] (LSA), in which we set the number of neighbours as 6 and the number of subspaces as c+1, or the number of classes plus one.
- RANSAC for Subspace Clustering [29] (RAN), in which we use the default settings.
- Our Projected Affinity Values on all non-zero eigenvectors (PAV+), in which we project the empirical affinity vector *k* onto all non-zero eigenvectors.
- Our Projected Affinity Values on *c* eigenvectors (PAV), in which we project *k* onto only *c* leading eigenvectors.

The ground-truth labels of data are used as the benchmark for all algorithms for comparison. We use the clustering accuracy [30] and Normalized Mutual Information (NMI) [31] to evaluate the clustering results of all algorithms, although many other metrics are available [32].

Accuracy is defined as:

(20) $$\mathrm{Accuracy}=\frac{\sum_{i=1}^{n}\delta ({\hat{c}}_{i},\mathrm{map}\left(a_{i}\right))}{n}\times 100$$

where ${\hat{c}}_{i}$ is the true label and $a_{i}$ is the derived label of the *i*-th data; δ(p,q) is the delta function where δ(p,q)=1 if p=q and δ(p,q)=0 otherwise; and map(·) is the best mapping function that matches the true labels and the derived labels. A larger value of accuracy indicates a better clustering performance. Accuracy is known to be improper for evaluation if data are unbalanced. Therefore, we also show in Table 1 the ratio of class sizes the maximum vs. the minimum. As shown in Table 1, the most unbalanced case occurs on Ionosphere with a ratio of 1.7857. Since there is not a significant unbalanced data distribution occurring in our employed datasets, we use accuracy to evaluate the clustering performance.

NMI is the second performance measure used in this paper. Let *M* and *N* be the random variables represented by the clustering labels generated by two competing methods. Denote by I(M,N) the mutual information between *M* and *N* and by H(M) the entropy of *M*. Then, NMI is defined as:

(21) $$\mathrm{NMI}\left(M,N\right)=\frac{I(M,N)}{\sqrt{H(M)H(N)}}$$

NMI ranges from 0–1 and takes the unitary value when two clustering labels are perfectly matched.

In our experiments, we adopt Gaussian kernel $\ker\left(x,y\right)=\exp\left(-∥x-y{∥}^{2}/2\sigma^{2}\right)$ in calculating the affinity matrix of a dataset. Although there are several σ adaptive selection methods [33,34], in general, the selection of a proper σ is a challenging problem. In our experiments, we set the Gaussian scale parameter σ as the square root of the average distance among the data. In the performance evaluation, we compare all methods with the ground-truth labels.

### 4.2. Real-World Dataset Experiment

In this section, we employ the competing methods on several benchmark datasets in clustering tasks. We run each method 100 times and report the average values in Table 2 and Table 3. We also list the average time cost in Table 4. In Table 4, we show the time cost in seconds of the standard Nyström approximation, which serves as a benchmark method in our test. Then, we show the time cost ratio, compared with Nyström, in Table 4, where 10× indicates a ten-times higher running time compared with Nyström. Several results are missing due to a failure to complete clustering or speed concerns.

From Table 2 and Table 3, we observe the following:

(1) Nyström-based clustering methods, namely the standard Nyström, our proposed PAV+ and PAV, show comparable or lower performances than the subspace pursuit methods (SSC, LSA and RAN) in terms of accuracy and NMI. A set of carefully selected subspaces that are suitable for clustering was identified in previous work and is shown in Table 2 and Table 3. However, as shown in Table 4, such advantages are always accompanied by additional time costs, which, in most cases, makes such approaches at least one order of magnitude slower than Nyström methods.
(2) SSC outperforms the other subspace-based methods. On the Wine dataset, SSC obtains much higher accuracy than any other method, and on Ionosphere, SSC obtains the highest NMI. The highest performance of SSC has been verified on many clustering tasks, particularly if one dataset can be sparsely self-represented. A sparse representation in SSC reveals the subspaces on which the classes lie. However, if a dataset is not sparse, the self-representation matrix *C*, as the substitute for the similarity matrix in spectral clustering, is more similar to a dense matrix. In this case, SSC may show similar performance to the traditional spectral clustering methods.
(3) Projection on all non-zero eigenvectors, or PAV+, shows similar results compared with the standard Nsytröm method. In contrast, once we project *k* onto the leading *c* eigenvectors, the corresponding results are slightly better than both standard Nyström and PAV+. The proposed PAV method, or using $k^{*}$ instead of *k* in Nyström, improves the clustering performance.
(4) The proposed method obtains a narrow margin over others in term of accuracy. Compared with the standard Nyström, we only replace *k* with $k^{*}$, and such changes may be very limited if the leading eigenvectors are well structured. However, considering the also very limited additional time burden introduced by ours, a slight improvement over the standard one is still a promising solution to Nyström.
(5) Nyström-based methods run much faster than subspace-pursuit methods perform. Among the three Nyström methods, the proposed PAV incurs limited additional time cost and, in return, obtains higher accuracy and NMI. Compared with SSC, the proposed PAV achieves comparable and occasionally superior performance with a running time that is at least one order of magnitude shorter. SSC needs to solve the sparse representation matrix *C* of data, or equivalently solving the $L_{1}$ optimization of input data. Such optimization is known to be time consuming. LCA requires additional local sampling that takes time to build a sparse connection matrix. RAN repeatedly samples a small subset of points, and such loops require a long running time. In contrast, the standard Nyström randomly samples the training data points and solves the eigenvectors of the small training set, a process with low computational burden. Our proposed method requires similar time consumption as that of Nyström, as discussed in Section 3.4. Thus, the time costs of Nyström and ours are much lower than those of SSC, LSA and RAN.

**Figure 2:**
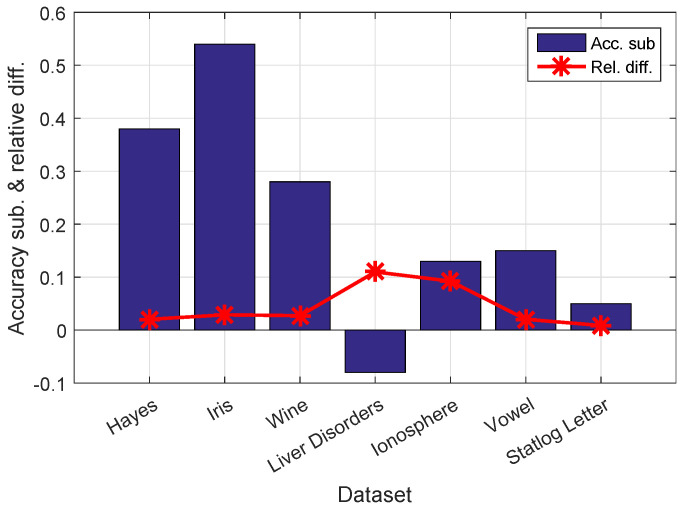
Accuracy subtraction and corresponding relative affinity difference. We subtract the accuracy value of the standard Nyström from that of our proposed method and then calculate the corresponding relative affinity difference, namely, $∥k-k^{*}∥/∥k∥$. Our replacement of *k* by $k^{*}$ constitutes a maximum relative difference of 10.99% and a minimum of 0.87% among the seven datasets.

Our main contribution is to replace *k* with $k^{*}$. In this test, we calculate the relative difference between *k* and $k^{*}$, namely, $\mathrm{diff}=∥k-k^{*}∥/∥k∥$, on each dataset. The relative differences for all seven datasets are shown in Figure 2. In addition, we subtract the accuracy of standard Nyström from that of our proposed method and show the results in Figure 2.

By projecting *k* onto leading eigenvectors, the relative affinity difference between *k* and $k^{*}$ ranges from 0.87% on Statlog Letter to 10.99% on Liver Disorders. In return, our $k^{*}$ improves the accuracy values on six of the seven datasets. The proposed method obtains an average improvement of 0.21 over all datasets, and the average difference is 4.4%. Our method improves accuracy even with a slight change of *k*. For example, on the Iris dataset, our $k^{*}$ differs by only 2.89% from *k*, but the accuracy improvement is 0.54.

### 4.3. Various Values of Gaussian Scale Parameter σ

**Figure 3:**
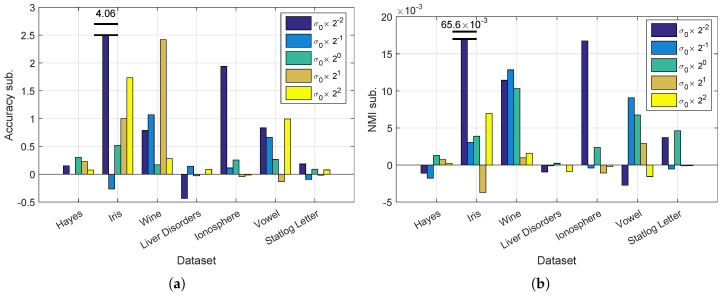
Clustering performances for various values of σ. For each value of σ, we subtract the Accuracy and NMI of standard Nyström from those of our method on all seven testing datasets. (**a**) Accuracy; (**b**) NMI.

In the Gaussian kernel, σ is a key parameter for clustering. The selection of σ is a difficult problem and always demands careful consideration. In this experiment, we test the performance of our method with various values of σ. We set $\sigma =\sigma_{0}\times 2^{\left[-2,\ldots ,2\right]}$, where $\sigma_{0}$ is the square root of the average distance among the data as used in previous experiments. We show in Figure 3 the clustering performances for different values of σ. Similar to our previous experiments, we subtract the performance value, i.e., accuracy or NMI, of Nyström from that of our method and show the results in Figure 3. On the Iris dataset with $\sigma =\sigma_{0}\times 2^{-2}$, the proposed method obtains improvements of 4.06 for accuracy and 0.065 for NMI; both values are much higher than their counterparts. For a better visualization, the corresponding bars are truncated.

In Figure 3, the proposed $k^{*}$ achieves the largest improvements in accuracy of 4.06 and NMI of 0.065 on Iris with $\sigma =\sigma_{0}\times 2^{-2}$. In the worst case, our method decreases accuracy by 0.43 on Liver Disorders and NMI by 0.004 on Iris. Our method shows average improvements of 0.45 in accuracy and 0.003 in NMI.

As σ changes, the structure of *K* may also change accordingly, and so of its eigenvectors. We assume that the leading eigenvectors of *K* and the spanned eigenspace that our $k^{*}$ projected to are more robust than the original *K*. Such robustness ensures a stable clustering even with an improper σ defined by users.

### 4.4. Various Training Set Sizes

**Figure 4:**
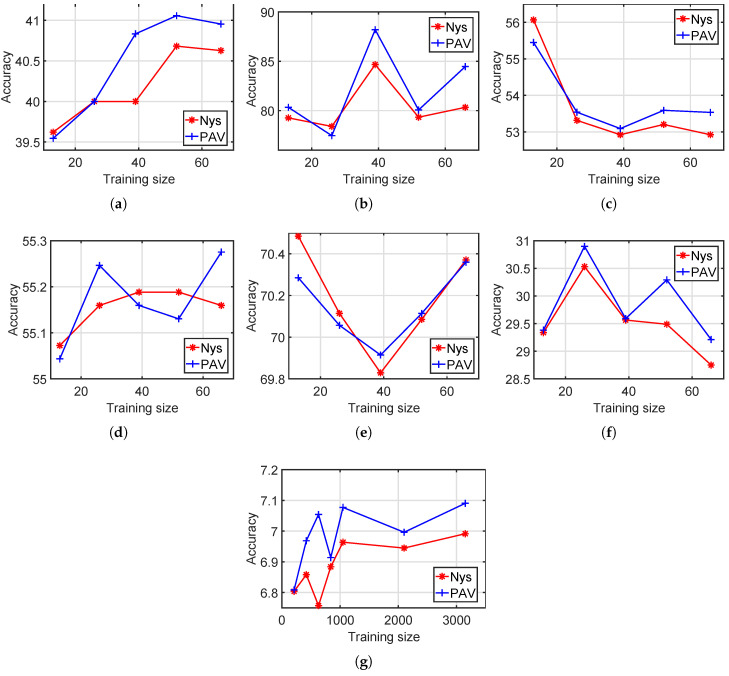
Clustering performance with various training set sizes, accuracy. (**a**) Hayes; (**b**) Iris; (**c**) Wine; (**d**) Liver Disorders; (**e**) Ionosphere; (**f**) Vowel; (**g**) Statlog Letter.

**Figure 5:**
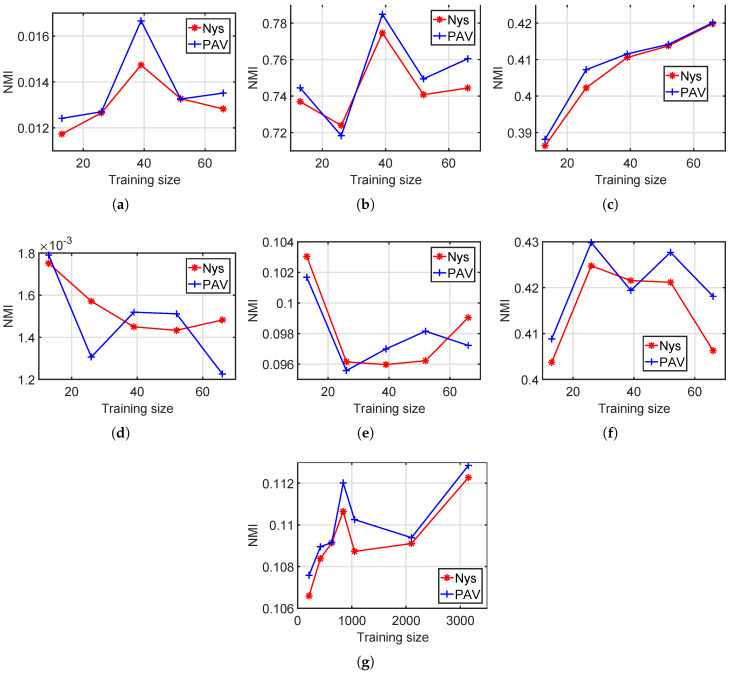
Clustering performance with various training set sizes, NMI. (**a**) Hayes; (**b**) Iris; (**c**) Wine; (**d**) Liver Disorders; (**e**) Ionosphere; (**f**) Vowel; (**g**) Statlog Letter.

In this experiment, we test both standard Nyström and our method with various training set sizes. We set the training set sizes for the six small datasets, namely, Hayes, Iris, Wine, Liver Disorders, Ionosphere and Vowel, with s=[0.1,0.2,…,0.5]×datasize. On Statlog Letter, we set the training set size as s=[0.02,0.04,…,0.1,0.2,0.3]×datasize. We run each method 10 times and show the average performances in Figure 4 and Figure 5.

According to Figure 4 and Figure 5, for most training set sizes, the proposed PAV performs slightly better than the standard Nyström. For example, on Statlog Letter, the average accuracy among all seven training sizes is 6.88 for Nyström and 6.99 for our method. The average NMI values of Nyström and our PAV are 0.1093 and 0.11, respectively, on Statlog Letter.

In Figure 4 and Figure 5, there are roughly two types on clustering results with respect to the training size: (a) better performance with more training data and (b) fairly unchanged. It is expected in general that more training points will improve the clustering results, as shown in Figure 4a. If one dataset has a clear structure suitable for clustering, then the potential improvement from increasing training points is limited since a small training set, in this case, is already sufficient enough for a good partition. Thus, clustering performances of increasing training points are fairly unchanged in Figure 4c–f.

### 4.5. Large-Scale Embedding

**Figure 6:**
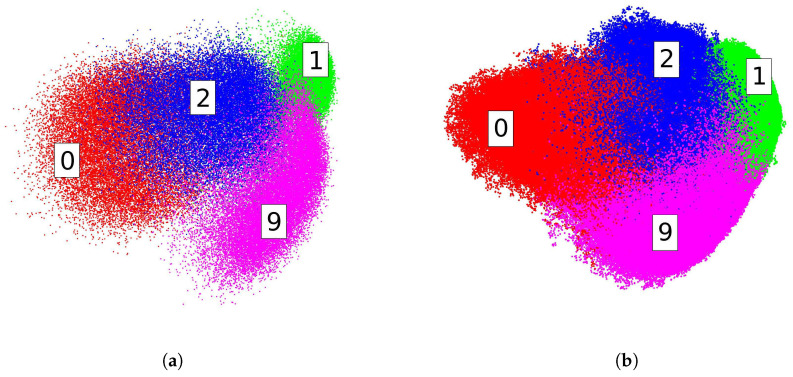
Embeddings for Digits 0, 1, 2 and 9 of MNIST-8M on the leading two dimensions. (**a**) EMNIST-Digits and (**b**) MNIST-8M.

In this experiment, we test the embedding performances of our method on two large-scale datasets. We evaluate our method on EMNIST-Digits and MNIST-8M. MNIST-8M contains 8.1 M data points, which are constructed by the elastic deformation of the original MNIST training set. To facilitate visualization, we only employ Digits 0, 1, 2 and 9, which yields a subset of approximately 3.3 M data points. We limit our test to 0, 1, 2 and 9 on EMNIST-Digits for the same reason. Figure 6 shows our embedding results on the first two dimensions with s=1200. Digits in the embedding space are well structured. In this test, we use two GeForce GTX TITAN Black GPUs with 6 GB memory for acceleration. The running time of MNIST-8M is 25.68 s and 9.84 s for EMNIST-Digits.

## 5. Conclusions

In the Nyström approximation on the Gaussian kernel, we suggest replacing the commonly-used affinity vector *k* with its projections on the leading eigenvectors that are learnt on the training set. Our work is motivated by the constraints that the new data points in the kernel space (a) have a close affinity value specified by *k* and (b) are on the unit sphere, as required by the Gaussian kernel. This optimization process is identical to the quadratic optimization over a sphere problem, and we prove in this paper that the Nyström approximation on the Gaussian kernel corresponds to the degeneracy case in the QOOS problem. Then, we show that, $k^{*}=\sum_{i=1}^{c}k^{T}u_{i}u_{i}$ has a corresponding $X_{new}$ that optimizes the QOOS problem. Thus, we suggest replacing *k* with $k^{*}$ in Nyström-based clustering. The experimental results on several real-world datasets verify the advantages of our proposed method over several popular methods.

References

## Figures and Tables

**Table 1:** Summary of datasets.

Type	Dataset	Size	Dimensions	Classes	Class Max./Min.
Small	Hayes	132	5	3	51/30
	Iris	150	4	3	50/50
	Wine	178	13	3	71/48
	Liver Disorders	345	6	2	200/145
	Ionosphere	351	34	2	225/126
	Vowel	528	10	11	48/48
	Statlog Letter	10,500	16	26	456/372
Large (embedding only)	EMNIST-Digits	240,000	784	10	24,000/24,000
	MNIST-8M	8,100,000	784	10	910,170/731,835

**Table 2:** Comparisons on real-world datasets, accuracy. Nys: Nyström; SSC: Sparse Subspace Clustering; LSA: Local Subspace Analysis; RAN: RANSAC for Subspace Clustering; PAV+: Our Projected Affinity Values on all non-zero Eigenvectors; PAV: Our Projected Affinity Values on *c* eigenvectors.

Dataset	Nys	SSC	LSA	RAN	PAV+	PAV
Hayes	40.53	37.12	38.64	38.64	40.53	40.91
Iris	78.13	78.47	39.95	43.60	78.13	79.73
Wine	53.29	66.85	43.74	53.10	53.29	53.76
Liver Disorders	55.87	59.71	52.46	56.06	55.14	55.79
Ionosphere	70.03	70.66	62.96	65.35	70.01	70.16
Vowel	29.63	25.60	21.95	- ${}^{1}$	29.49	29.78
Statlog Letter	6.86	-	-	-	6.87	6.91

${}^{1}$ indicates missing results due to a failure to complete clustering or speed concerns.

**Table 3:** Comparisons on noised real-world datasets, Normalized Mutual Information (NMI).

Dataset	Nys	SSC	LSA	RAN	PAV+	PAV
Hayes	0.01	0.01	0.02	0.01	0.01	0.02
Iris	0.71	0.59	0.02	0.05	0.72	0.72
Wine	0.43	0.37	0.09	0.19	0.42	0.45
Liver Disorders	0.00	0.03	0.00	0.01	0.00	0.00
Ionosphere	0.10	0.15	0.03	0.04	0.10	0.10
Vowel	0.42	0.32	0.25	- ${}^{1}$	0.42	0.43
Statlog Letter	0.11	-	-	-	0.11	0.11

${}^{1}$ indicates missing results due to a failure to complete clustering or speed concerns.

**Table 4:** Comparisons on real-world datasets, time cost in s or as a ratio.

Dataset	Time Cost (s)	Time Cost (Ratio)				
	Nys	SSC	LSA	RAN	PAV+	PAV
Hayes	0.0093	36.0×	41.7×	65.1×	1.0×	1.0×
Iris	0.0112	20.8×	42.6×	1.1×	1.5×	1.0×
Wine	0.0123	19.9×	55.5×	69.1×	1.4×	1.0×
Liver Disorders	0.0449	13.2×	49.0×	9.0×	1.4×	1.0×
Ionosphere	0.0251	49.0×	93.6×	104.7×	1.8×	2.3×
Vowel	0.0794	25.5×	66.5×	- ${}^{1}$	2.0×	1.0×
Statlog Letter	2.8828	-	-	-	1.4×	1.4×

${}^{1}$ indicates missing results due to a failure to complete clustering or speed concerns.
